# Improving the Safety of Tourniquet Use in a Trauma Theatre According to the British Orthopaedic Association Guidelines: A Closed Loop Audit

**DOI:** 10.7759/cureus.51601

**Published:** 2024-01-03

**Authors:** Sushanth Vayalapra, Daniel N Guerero, Balakumar Balasubramanian, Prakash Palaparthy, Mohanraj Venkatesan, Maneesh Sinha

**Affiliations:** 1 Trauma and Orthopaedics, Russells Hall Hospital, Dudley, GBR

**Keywords:** surgical documentation, boast, retrospective audit, orthopaedic traumatology, tourniquet use

## Abstract

Introduction

Tourniquets are used widely in trauma and orthopaedic surgery to reduce blood loss and facilitate better visualisation of the operative field; however, some complications can result from improper use such as pressure sores, chemical burns, compartment syndrome, and deep vein thrombosis. We audited the use of intraoperative tourniquets in our trauma theatre against the guidance published by the British Orthopaedic Association (BOA) in 2021.

Methods

This was a closed-loop audit evaluating 80 trauma operations that utilised tourniquets. In the first cycle, we audited 40 operations (23 upper limbs vs 17 lower limbs) over a period of two months through a review of operation notes and theatre documentation. We presented our findings and implemented changes including the addition of tourniquet use to the operation note template and labels on the tourniquet machines aiding the calculation of tourniquet pressures. A re-audit was then performed involving a further 40 operations (20 upper limbs and 20 lower limbs). Statistical analyses were performed to compare the two cycles.

Results

Tourniquet time was on average similar across both audit cycles (60.7 vs 70.0, *p* = 0.192) with compliance up to standard in 97% of cases. Post-intervention, there was an improvement in the documentation of skin status (37 vs 69%, *p *= 0.004), tourniquet isolation method (43% vs 74%, *p *= 0.003), and tourniquet pressure (71% vs 94%, *p *= 0.003). The difference between tourniquet pressure and systolic blood pressure was on average lower post-intervention for the upper limb (125.9 vs 99.9, *p* < 0.01) and lower limb operations (154.2 vs 121.7, *p *< 0.01). Adherence to the British Orthopaedic Association Standards for Trauma (BOAST) guidance with tourniquet pressure improved with intervention (25% vs 75%).

Conclusion

The introduction of tourniquet parameters in the operation note template and patient-specific calculation of tourniquet pressures improved the safe use of tourniquets within the trauma theatre.

## Introduction

A tourniquet is any device applied externally to a body part to compress and occlude underlying vascular structures to reduce blood flow. The pressure applied by the tourniquet must be greater than the arterial blood pressure to stop the blood flow successfully. The minimal pressure required to stop arterial inflow to a limb is termed the limb occlusion pressure (LOP) or the arterial occlusion pressure (AOP) [1]. LOP is measured through the stepwise inflation of a pressure cuff until the loss of the distal arterial pulse on Doppler ultrasound scanning or pulse oximetry [2]. AOP can be approximated using a formula based on a patient’s systolic blood pressure (SBP) and tissue padding coefficient [3,4].

Intraoperative tourniquets are used to reduce bleeding, which results in a clearer operative field of view. Tourniquet use has also been associated with significant reductions in operative times with certain orthopaedic procedures [5]. Despite these benefits, tourniquet use is not without its own risks. It has been associated with complications such as nerve injury [6], chemical burns [7], deep venous thrombosis or pulmonary embolism [8], tourniquet pain [9], rhabdomyolysis [10], and compartment syndrome [11]. The incidence and severity of tourniquet-related complications are directly related to tourniquet insufflation pressures [12] and prolonged tourniquet use [9].

The British Orthopaedic Association Standards for Trauma (BOAST) guidelines on “the safe use of intraoperative tourniquets” provide a series of recommendations on the safe limits for tourniquet time and pressure [13]. These guidelines use the SBP as a proxy for LOP as the measurement of the LOP is less practical in clinical practice. The SBP is used alongside a set pressure based on the patient’s age and location of surgery to calculate the final tourniquet pressure. This helps minimise the overinflation of the tourniquet and its potential complications. The BOAST guidelines also stress the importance of documenting tourniquet use in operative procedures. Clear documentation helps keep a record of several factors such as how the tourniquet is isolated which are critical in minimising the risk of adverse effects [14]. The record of tourniquet use is also useful for audit, research [15], and medico-legal purposes [16].

This audit aimed to improve compliance with these recommendations in the Trauma and Orthopaedic Department of a District General Hospital. The decision regarding tourniquet pressure was made by the operating surgeon at the start of the procedure. The department developed a culture of using standard tourniquet inflation pressures of 250 mmHg for upper limb cases and 300 mmHg for lower limb surgeries independent of patient age, weight, comorbidities, and blood pressure. Similar practices have been previously reported [2]. The use of these pre-set insufflation pressures can often be excessive and therefore expose patients to a greater and unnecessary risk of developing tourniquet-related complications. This project aimed to reduce this risk while still ensuring effective tourniquet use.

## Materials and methods

This retrospective audit was conducted within the trauma and orthopaedic department of a local District General Hospital. It was registered with the hospital trust’s clinical audit team as an audit project with the project code T&O/CA/2022-23/19.

The standard that was employed for the audit was the BOAST guidelines on the safe use of intraoperative tourniquets [13]. The guidelines list several parameters that should be met when using tourniquets in theatres, which are shown in Table 1.

**Table 1:** Modified BOAST guidelines on the safe use of intraoperative tourniquets [13] BOAST: British Orthopaedic Association Standards for Trauma.

Audit standard parameters
Documentation of the condition of the tourniquet site prior to and at the end of the procedure
Documentation of the method of isolation used to exclude skin preparation fluids from seeping under the tourniquet
Patients < 16 years should have a tourniquet pressure of limb occlusion pressure plus 50 mmHg or systolic blood pressure plus 50-100 mmHg
Patients > 16 years should have a tourniquet pressure of systolic blood pressure plus 70-130 mmHg for the lower limb and 50-100 mmHg for the upper limb
The ischaemic time should be less than 120 minutes

The first cycle was conducted between September 2022 and October 2022, and the second cycle was performed between February 2023 and March 2023. All upper and lower limb operations that involved the use of a tourniquet over two months were included as part of the study. Elective operations and operations which used finger or toe tourniquets were excluded.

Data was collected from the physical operative logbook within the trauma theatre as well as the electronic operation note documentation. This included the patient’s age, sex, operative procedure, indication for tourniquet use, tourniquet site documentation, isolation method, SBP at the time of inflation, tourniquet pressure, and tourniquet time. The analysis was performed using Microsoft Excel version 16 (Microsoft Corporation, Redmond, WA) and SPSS for Mac version 29 (IBM Corp., Armonk, NY). A Shapiro-Wilk test was carried out to assess the normal distribution of the data. For statistical analyses, Pearson chi-square test, independent samples t-test, and Mann-Whitney U test were used.

**Figure 1:**
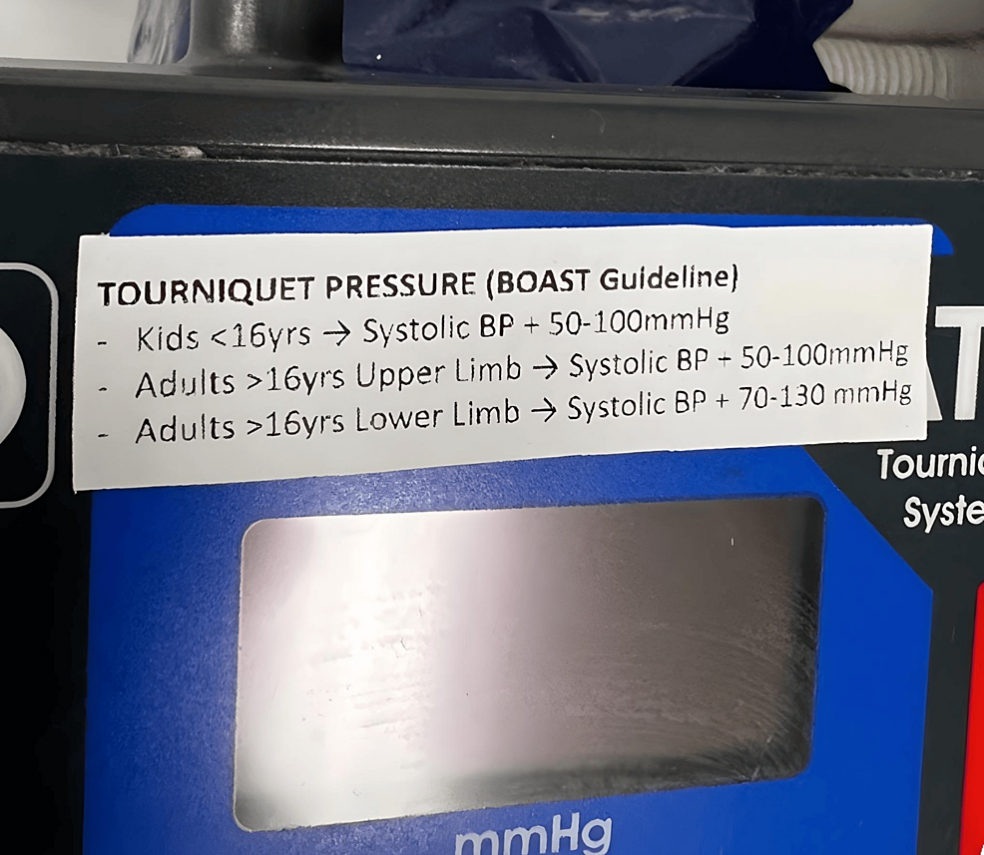
Label applied to tourniquet machine explaining the calculation of tourniquet pressure

Before the second audit cycle, the data was presented at the local trauma and orthopaedic departmental audit meeting. Interventions introduced included the addition of tourniquet details as a mandatory component of the electronic operation note template. Labels were also applied to the tourniquet machines to aid theatre staff in the calculation of appropriate tourniquet pressures (see Figure 1). A copy of the BOAST guidance was printed and applied to the wall in the trauma theatre doctor’s office as a reminder for both trainee and consultant surgeons when completing operative documentation.

## Results

The patient demographics are outlined in Table 2. It shows similar characteristics between the patients and operative cases across both audit cycles.

**Table 2:** Patient demographics

	Audit	Re-audit	*p*-value
No. of patients	40	40	
M:F ratio	21:19	18:22	0.811
Age			
Mean (SD)	53 (23)	48 (20)	0.134
Range	10-92	9-87	
Upper limb vs lower limb	23 vs 17	20 vs 20	0.501
Operation type			
Open reduction internal fixation	26	29	
Wound debridement	5	4	
Tendon repair	3	2	
Ligament repair	2	1	
Arthroscopic washout	3	2	
Incision and drainage of abscess	1	2	

Overall, documentation of skin status, isolation method, and tourniquet pressure improved in the re-audit after intervention (Table 3).

**Table 3:** Rates of documentation for tourniquet parameters

	Audit	Re-audit	*p-*value
Tourniquet skin status documented before and after the procedure	37%	69%	0.004
Tourniquet isolation method documented in the operation note	43%	74%	0.003
Tourniquet pressure documented in the operation note	71%	94%	0.003

In both audit cycles, tourniquet time remained less than 120 minutes for 97% of operative cases with the maximum tourniquet time recorded as 136 minutes. The tourniquet times were similar between the first and second audit cycles (independent samples t-test, mean: 60.7, SD: 35.4 vs mean: 70.0, SD: 27.9, *p* = 0.192).

**Figure 2:**
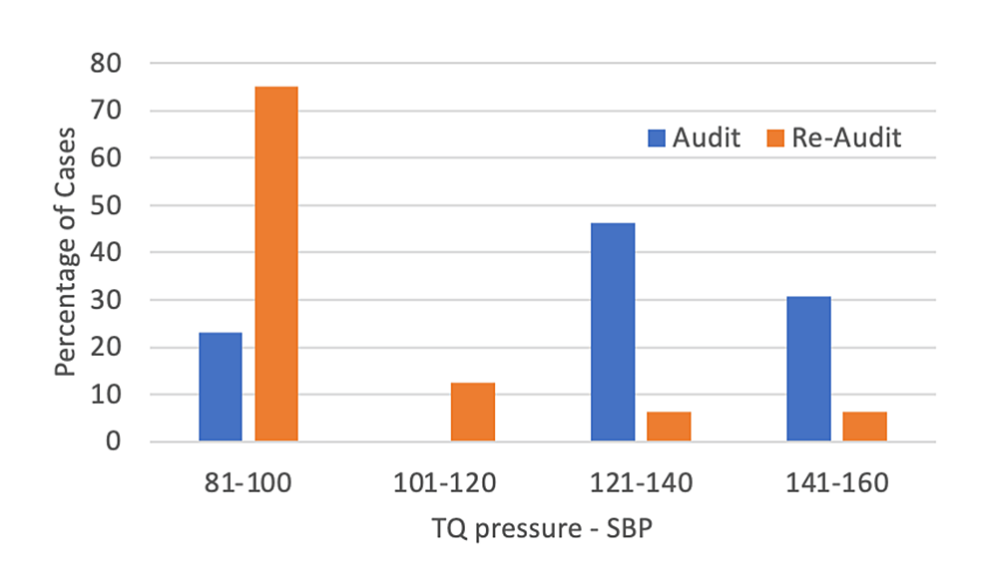
Difference between tourniquet pressure and systolic blood pressure in both audit cycles for upper limb operations. A range of 50-100 mmHg is the safe limit recommended by the BOAST guidance. BOAST: British Orthopaedic Association Standards for Trauma; SBP: Systolic blood pressure; TQ: Tourniquet.

**Figure 3:**
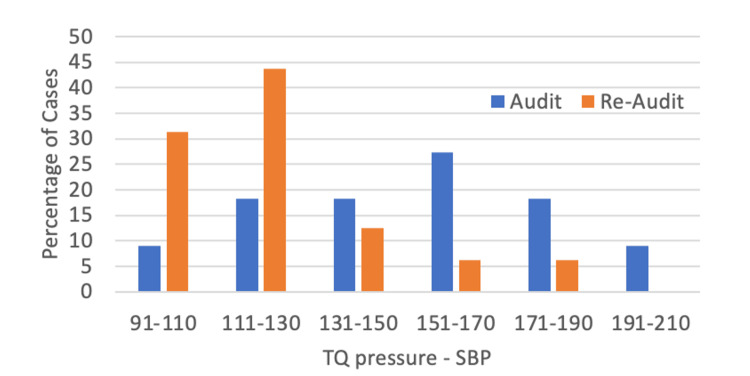
Difference between tourniquet pressure and systolic blood pressure in both audit cycles for lower limb operations. A range of 70-130 mmHg is the safe limit recommended by the BOAST guidance. BOAST: British Orthopaedic Association Standards for Trauma; SBP: Systolic blood pressure; TQ: Tourniquet.

The difference between SBP and tourniquet pressure was reduced in the re-audit cycle for the upper limb (Mann-Whitney U test, mean: 125.9, SD: 23.6 vs mean: 99.9, SD: 19.8, *p* < 0.01) and lower limb operations (Mann-Whitney U test, mean: 154.2, SD: 29.7 vs mean: 121.7, SD: 20.3, *p* < 0.01). Graphs outlining the difference between tourniquet pressure and SBP are shown in Figures 2, 3. About 75% of cases from the re-audit were operating within the safe limit described by the BOA compared to 25% in the first audit cycle.

## Discussion

Our closed-loop audit showed an improvement in compliance with the documentation of tourniquet use after multiple interventions were implemented. Documentation of skin status and tourniquet isolation method improved from 38% (n = 15) to 70% (n = 28) and 43% (n = 17) to 75% (n = 30) post-intervention, respectively. Tourniquet pressures were documented in 95% (n = 38) of cases in the re-audit cycle versus 70% (n = 28) in the first cycle. Our results were comparable to or better than those found at other centres. An audit of elective arthroplasty surgical notes across nine UK hospitals showed a compliance rate of 83% for the documentation of tourniquet time [17]. Other studies have noted particularly poor documentation rates for tourniquet use with compliance rates of 32% [18] and 42% [19].

The improvements noted in documentation rates were likely the result of making the tourniquet parameters a mandatory component of the electronic operation note template. A similar intervention has been trialled in another department where the introduction of a new time-efficient template has improved compliance by 49% [20]. Surgeons in our department who did not use the new template still had omissions in their documentation of tourniquet use.

Tourniquet time across both cycles met the BOAST standards in 97% of cases where the tourniquet remained inflated for less than the recommended two-hour limit. The average tourniquet time was comparable across both audit cycles and was significantly below the upper limit. This may reflect the lower complexity of the cases performed as this study was done in a District General Hospital as opposed to a Tertiary Referral Centre. The AT4 tourniquet machines have an alarm that sounds at 90 and 120 minutes as a safety mechanism to alert surgeons to the tourniquet time [21]. This has resulted in a generally high compliance with most cases remaining within the recommended time limit.

The use of standardised tourniquet pressures of 250 and 300 mmHg for upper and lower limb procedures, respectively, was widespread in our department as shown by the results of the first audit cycle. These standardised tourniquet pressures were consistent with findings from a survey of community-based and academic surgeons in the United States [22]. The lack of awareness of recent BOAST guidance on tourniquets was evident in our departmental meeting. Post-intervention, the difference between tourniquet pressure and SBP was lower, and the compliance rates improved by around 50%. About 25% of cases still used an insufflation pressure above the recommended range despite the interventions implemented. Compliance could be improved further by educating theatre staff on the appropriate calculation of tourniquet pressures and ensuring this is part of their mandatory training. The tourniquet machines could also be adapted to calculate tourniquet pressure based on the entry of the patient’s SBP.

With the widespread use of tourniquets, it is an assumption that surgeons and healthcare professionals working in the operating theatre understand how to use tourniquets safely to minimise complications. Prior studies and surveys have made it clear that this is not the case [23-25]. There is a general lack of formal training on tourniquet application with most trainees gaining skills in tourniquet application from more senior surgeons. This diversity in educational experience can be carried forward into clinical practice leading to a lack of consistency in tourniquet use. In the future, the integration of tourniquet application into the orthopaedic surgical curriculum may improve standards of care.

The limitations of our audit should be considered when interpreting the results. First, the data was collected from a single centre, limiting the generalisability of the findings. The limited sample size of 80 patients may not fully represent the diversity of practices with respect to tourniquet use. The faster-paced nature of the trauma theatre may have also led to poorer compliance rates when compared to those cases that were performed in the elective setting.

## Conclusions

This closed-loop audit has demonstrated that the implementation of a specific electronic operation note template and patient-specific calculation of tourniquet pressures alongside the active involvement of staff can lead to improvements in the safety of tourniquet use and adherence to best practice guidelines. We would encourage all orthopaedic units to evaluate their tourniquet use against BOAST guidance to improve the standards of care and minimise potential complications.

## References

[1] Kasem SA, Bassiouny AA, Rashwan DA, Bahr MH (2020). Minimal inflation tourniquet pressure using induced hypotension with limb occlusion pressure determination or arterial occlusion pressure estimation in upper limb surgery: a randomized double-blinded comparative study. Anesth Pain Med.

[2] Deloughry JL, Griffiths R (2009). Arterial tourniquets. Continuing education in anaesthesia, critical care and pain.

[3] Tuncalı B, Boya H, Kayhan Z, Araç Ş, Çamurdan MA (2016). Clinical utilization of arterial occlusion pressure estimation method in lower limb surgery: effectiveness of tourniquet pressures. Acta Orthop Traumatol Turc.

[4] Tuncali B, Karci A, Tuncali BE (2006). A new method for estimating arterial occlusion pressure in optimizing pneumatic tourniquet inflation pressure. Anesth Analg.

[5] Zaid HH, Hua X, Chen B, Yang Q, Yang G, Cheng W (2023). Tourniquet use improves intraoperative parameters, leading to similar postoperative outcomes compared with no tourniquet use in anterior cruciate ligament reconstruction: a prospective, double-blind, randomized clinical trial. Arthroscopy.

[6] Odinsson A, Finsen V (2006). Tourniquet use and its complications in Norway. J Bone Joint Surg Br.

[7] Yang JH, Lim H, Yoon JR, Jeong HI (2012). Tourniquet associated chemical burn. Indian J Orthop.

[8] Song JE, Chun DH, Shin JH, Park C, Lee JY (2010). Pulmonary thromboembolism after tourniquet inflation under spinal anesthesia-a case report. Korean J Anesthesiol.

[9] Kamath K, Kamath SU, Tejaswi P (2021). Incidence and factors influencing tourniquet pain. Chin J Traumatol.

[10] Türkmen İ, Esenkaya İ, Unay K, Akçal MA (2015). Rhabdomyolysis after tourniquet use in proximal tibial osteotomy: a case report and review of the literature. Acta Orthop Traumatol Turc.

[11] Hirvensalo E, Tuominen H, Lapinsuo M, Heliö H (1992). Compartment syndrome of the lower limb caused by a tourniquet: a report of two cases. J Orthop Trauma.

[12] Olivecrona C, Ponzer S, Hamberg P, Blomfeldt R (2012). Lower tourniquet cuff pressure reduces postoperative wound complications after total knee arthroplasty: a randomized controlled study of 164 patients. J Bone Joint Surg Am.

[13] (2023). BOAST - the safe use of intraoperative tourniquets. https://www.boa.ac.uk/resource/boast-the-safe-use-of-intraoperative-tourniquets.html.

[14] Hamad F, Rossiter N (2023). Tourniquet use in trauma and orthopaedics, how and when: current evidence. Surgery.

[15] Ruckle DE, Chang AC, Wongworawat MD (2023). The effect of upper extremity tourniquet time on postoperative pain and opiate consumption. Hand (NY).

[16] Harrison WD, Narayan B, Newton AW, Banks JV, Cheung G (2015). Litigation costs of wrong-site surgery and other non-technical errors in orthopaedic operating theatres. Ann R Coll Surg Engl.

[17] Severn Audit and Research Collaborative in Orthopaedics (SARCO) (2016). Assessing the quality of operation notes: a review of 1092 operation notes in 9 UK hospitals. Patient Saf Surg.

[18] Sweed TA, Bonajmah AA, Mussa MA (2014). Audit of operation notes in an orthopaedic unit. J Orthop Surg (Hong Kong).

[19] Tuteja S, Tiwari A, Bhanushali J, Bagaria V (2022). Results of an audit of orthopaedic operation notes from a tertiary care centre: are we doing it right and can we do more?. Indian J Orthop.

[20] Fitzpatrick N, Arneill M, Wilson L (2022). 903 improving documentation of tourniquet use in a trauma unit in line with new BOAST guidelines. British Journal of Surgery.

[21] (2023). AT4 electronic tourniquet system. https://aneticaid.com/products/at4-electronic-tourniquet-system.

[22] Tejwani NC, Immerman I, Achan P, Egol KA, McLaurin T (2006). Tourniquet cuff pressure: the gulf between science and practice. J Trauma.

[23] Younger AS, Kalla TP, McEwen JA, Inkpen K (2005). Survey of tourniquet use in orthopaedic foot and ankle surgery. Foot Ankle Int.

[24] Kalla TP, Younger A, McEwen JA, Inkpen K (2003). Survey of tourniquet use in podiatric surgery. J Foot Ankle Surg.

[25] Sadri A, Braithwaite IJ, Abdul-Jabar HB, Sarraf KM (2010). Understanding of intra-operative tourniquets amongst orthopaedic surgeons and theatre staff--a questionnaire study. Ann R Coll Surg Engl.

